# *Klebsiella pneumoniae* SnebYK Mediates Resistance Against *Heterodera glycines* and Promotes Soybean Growth

**DOI:** 10.3389/fmicb.2018.01134

**Published:** 2018-06-01

**Authors:** Dan Liu, Le Chen, Xiaofeng Zhu, Yuanyuan Wang, Yuanhu Xuan, Xiaoyu Liu, Lijie Chen, Yuxi Duan

**Affiliations:** ^1^Nematology Institute of Northern China, Shenyang Agricultural University, Shenyang, China; ^2^Institute of Plant Protection, Liaoning Academy of Agricultural Sciences, Shenyang, China; ^3^College of Biological Science and Technology, Shenyang Agricultural University, Shenyang, China; ^4^College of Plant Protection, Shenyang Agricultural University, Shenyang, China; ^5^College of Sciences, Shenyang Agricultural University, Shenyang, China

**Keywords:** induced systemic resistance, plant growth-promoting bacteria, *Klebsiella pneumoniae*, *Heterodera glycines*, soybean

## Abstract

Soybean is an important economic crop that is often adversely affected by infection in the field with the soybean cyst nematode *Heterodera glycines*. Biological control is an eco-friendly method used to protect the crop against disease. The bacterium *Klebsiella pneumoniae* has been reported to protect rice from sheath blight and seedling blight, but its role in the control of nematode is unclear. In this study, the effect of *K. pneumoniae* SnebYK on the control of *H. glycines* was assessed. Potting experiment results showed that coating soybean seeds with *K. pneumoniae* SnebYK not only reduced the infection rate of *H. glycines* but also decreased the proportion of adult female nematodes. Field experiment results showed that *K. pneumoniae* SnebYK reduced both the number of *H. glycines* in soybean roots and the number of adult females. However, *K. pneumoniae* SnebYK caused low juvenile mortality in an *in vitro* assay. To further analyze the role of *K. pneumoniae* SnebYK in the inhibition of *H. glycines* infection, split root experiments were conducted. The results indicated that *K. pneumoniae* SnebYK controls *H. glycines* via induced systemic resistance, which reduces *H. glycines* penetration. *Klebsiella pneumoniae* SnebYK treatment also significantly increased the proportion of second-stage juveniles and decreased the proportions of third- and fourth-stage juveniles in the *H. glycines* population. Moreover, 48 h after inoculation with *H. glycines*, the expression levels of *PR1, PR2, PR5*, and *PDF1.2* were significantly higher in soybeans pretreated with *K. pneumoniae* SnebYK than in control soybeans. Interestingly, besides providing protection against nematodes, *K. pneumoniae* SnebYK fixed nitrogen, produced ammonia, solubilized phosphate, and produced siderophores, leading to well-developed root system and an increase in soybean seedling fresh weight. These results demonstrate for the first time that *K. pneumoniae* SnebYK not only promotes soybean growth but also inhibits the invasion and development of *H. glycines* by inducing systemic resistance.

## Introduction

Soybean (*Glycine max*) is an important crop worldwide due to its nutritive and commercial value. The soybean cyst nematode (*Heterodera glycines* Ichinohe) is a soil-borne plant-parasitic nematode that disturbs the root growth of soybean and causes the early yellowing of plants (Subbotin et al., 2010). As a result of the widespread distribution of *H. glycines* and its ability to reduce seed yield, *H. glycines* is considered the most harmful pest that endangers soybean yield globally (Koenning and Wrather, 2010; Rincker et al., 2017). Depending on the severity of the infection, *H. glycines* generally decreases soybean production by 5–100% in northeast China, a major soybean production region (Duan, 2011). Meanwhile, in the United States, the loss caused by *H. glycines* has been estimated to exceed one billion USD annually (Koenning and Wrather, 2010). Thus, finding a simple, eco-friendly, and effective approach to control *H. glycines* is imperative for modern agriculture.

The use of plant growth-promoting bacteria (PGPB) is an ideal approach for the prevention and control of nematodes. PGPB promote plant growth and also induce systemic resistance (Wei et al., 1996; Kloepper et al., 2004). Induced systemic resistance (ISR) is a phenomenon whereby resistance to an infectious disease is systemically induced by localized infection, by treatment with microbial components or products or with a diverse group of structurally unrelated organic and inorganic compounds (Kuć, 2001). Jasmonic acid (JA)/ethylene (ET), and/or salicylic acid (SA) signaling pathways are important in the regulation of beneficial microbes-mediated ISR (Pieterse et al., 2014; Vryzas, 2016). In recent years, ISR mediated by PGPB (e.g., *Bacillus* sp., *Bacterium* sp., *Methylomonas methanica, Rhizobium etli*) has been intensively studied against a wide range of important plant-parasitic nematodes, such as *Meloidogyne incognita* (Anter et al., 2014; Alfianny et al., 2017), *H. glycines* (Xiang et al., 2013), and *Globodera pallida* (Reitz et al., 2000). Tian et al. (2014) reported that *Sinorhizobium fredii* decreased the infection rate of *H. glycines* by triggering ISR and also prolonged the developmental stage of *H. glycines* in the root to 30 days, compared with 27 days in the control. Xiang et al. (2013) also reported that treating soybean seeds with *B. simplex* reduced the *H. glycines* population by triggering ISR. Therefore, seed coating with PGPB is a simple and cost-effective strategy that elicits ISR in host plants against pathogens (Van Loon et al., 1998; Ramamoorthy et al., 2001; Pathak et al., 2016). After seed coating, these plants acquire broad-spectrum resistance without any changes to their genome sequences, and respond more rapidly and strongly to various stresses (Pathak et al., 2016).

*Klebsiella pneumoniae* is a strain of the PGPB that promotes plant growth by fixing nitrogen (Iniguez et al., 2004); producing 1-aminocyclopropane-1-carboxylate deaminase (Singh et al., 2015), indole-3-acetic acid (Sachdev et al., 2009), gibberellic acid (Singh et al., 2015), and siderophores; and solubilizing phosphate (Ji et al., 2014). *Klebsiella* sp. KW7-S06 elicited ISR to the pathogenic fungi *Fusarium oxysporum* and *Rhizoctonia solani* in rice, alleviating disease symptoms (Ji et al., 2014). However, reports of *K. pneumoniae*-elicited ISR to nematodes are few. Furthermore, finding a candidate biocontrol agent against *H. glycines* would have great significance for the soybean industry. In the present study, we aimed to (1) assess the plant growth-promoting properties of the *K. pneumoniae* strain SnebYK and its effect on soybean growth, (2) evaluate the effect of the *K. pneumoniae* strain SnebYK on the control of *H. glycines* via seed coating, and (3) investigate the ability of the *K. pneumoniae* strain SnebYK to induce systemic resistance to *H. glycines* in soybean.

## Materials and Methods

### Strain, Fermentation Broth, and Nematode

*Klebsiella pneumoniae* strain SnebYK, subsequently referred to as SnebYK, is a Gram-negative facultatively anaerobic bacterium that was isolated from mud at the outlet of a pesticide manufacturing factory in Liaoning Province, China. SnebYK was identified by Luo (2010) and stored at -80°C at the Nematology Institute of Northern China at Shenyang Agricultural University. Before use, the strain was streaked onto a nutrient agar medium and incubated at 28°C to confirm purity. A single colony was selected, transferred to nutrient agar medium, and incubated for 48 h at 28°C. The SnebYK fermentation broth (1 × 10^9^ CFU ml^-1^) was prepared according to the method previously described by Chen et al. (2014).

The population of *H. glycines* (race 3, HG type 0) was maintained on soybean [*Glycine max* (L.) Merr. ‘Liaodou15’] grown in the experimental field of the Nematology Institute of Northern China. *Heterodera glycines* specimens were morphologically identified following the method previously described by Subbotin et al. (2010) prior to use in the experiment. Cysts were extracted from soybean rhizosphere soil in the experimental field using a floating sieve, and then surface-sterilized with 0.5% sodium hypochlorite. Second-stage juveniles (J2) were incubated as described by Tian et al. (2014). The hatched J2 were assessed for survival and collected in a beaker for inoculation.

### Biochemical Assays of Putative Plant Growth-Promoting Properties

The siderophore production of SnebYK was determined as described by Schwyn and Neilands (1987). SnebYK was inoculated in the center of a chrome azurol S agar plate and incubated at 28°C for 7 days; plates were observed to determine whether a yellow–orange halo appeared around the colony. Assays of inorganic and organic phosphate solubilization by SnebYK were performed using the National Botanical Research Institute’s phosphate growth medium and Mongina organic culture medium with lecithin, respectively (Hao et al., 2012; Ji et al., 2014). SnebYK was spot-inoculated onto the plate and incubated at 28°C for at least 7 days. A clear zone around the colony indicated that the strain tested had phosphate solubilization activity. The phosphate-solubilizing ability of SnebYK was evaluated using the phosphomolybdate blue colorimetric method (Murphy and Riley, 1962). Ammonia production was detected with Nessler’s reagent (Singh et al., 2015).

The nitrogen-fixing ability of SnebYK was tested using ACCC55 agar plates (Gao et al., 2015). The nitrogenase activity of SnebYK was evaluated through an acetylene reduction assay, following the protocol of Boddey and Knowles (1987). To analyze the *nifH* gene of SnebYK, genomic DNA was extracted from the SnebYK strain using a bacterial genomic DNA extraction kit (Tiangen Biotech, China). The primers nifH1 (5′-ADNGCCATCATYTCNCC-3′) and nifH2 (5′-TGYGAYCCNAARGCNGA-3′) were used for polymerase chain reaction (PCR) amplification of the *nifH* gene (Gaby and Buckley, 2012). The PCR amplification conditions were 5 min at 94°C for initial denaturation; 35 cycles of 94°C for 1 min, 57°C for 1 min, and 72°C for 1 min, followed by a 72°C extension for 10 min (Liu et al., 2011). PCR products were analyzed by electrophoresis on 1.2% agarose gels and then sequenced by Sangon Biotech Co. Ltd., Shanghai, China. The obtained sequences were compared with those in the GenBank database (**RRID**: *SCR_002760*) using the NCBI BLAST algorithm (Pinto-Tomás et al., 2009; Liu et al., 2011; Lin et al., 2012). The phylogenetic tree was established using the neighbor-joining method in Mega 7.0.26. To detect the expression of *nifH*, reverse transcription (RT)-PCR analysis was conducted. SnebYK colonies grown on ACCC55 agar plates were collected, and colonies grown on nutrient agar plates were also collected as controls. Total RNA was extracted from the samples using a bacterial total RNA extraction kit (Tiangen Biotech, China), and the PrimeScript RT Reagent kit (TaKaRa, China) was used for cDNA synthesis following the manufacturer’s instructions. The primers used to amplify *nifH* were PolF (5′-TGCGAYCCSAARGCBGACTC-3′) and PolR (5′-ATSGCCATCATYTCRCCGGA-3′) (Poly et al., 2001). The 16S rRNA gene was used as an internal control. For the PCR, 100 ng cDNA was used, with the following program: 94°C for 4 min; 30 cycles of 94°C for 30 s, 60°C for 30 s, and 72°C for 30 s, followed by an extension at 72°C for 5 min. PCR products were visualized using the GoldView DNA staining reagent (Aidlab Biotech, China) following agarose gel electrophoresis and analysis.

### Growth Promotion Assay in Soybeans Treated With SnebYK

Soybean seeds were surface-sterilized with 0.5% sodium hypochlorite, washed with sterilized water at least three times, and then dried at room temperature. Treated soybean seeds were coated with the SnebYK fermentation broth at a 70:1 mass ratio. Control soybean seeds were coated with sterilized water. All seeds were sown in 10 cm × 10 cm plastic pots containing a sterilized mixture of soil and sand (1:1, *v/v*) and grown in a greenhouse at 26/21°C with a 16/8 h photoperiod. The soybean seedlings were thinned to one plant per pot once two euphylla were observed. Each treatment included 10 replicates and was arranged in a completely randomized design. Thirty days after sowing, soybean seedlings were harvested from all pots, the soil adhered to the roots was carefully removed with tap water, and the shoot length, taproot length, and fresh weight of the plants in each treatment were measured. Root morphology images were obtained using a scanner (Expression 10000 XL, Epson, Japan) and then analyzed using the WinRHIZO image analysis system (V4.1c, Regent Instruments, Canada).

### Effect of SnebYK on *H. glycines* Control

#### Juvenile Mortality *in Vitro* Assay

To obtain the culture filtrate, SnebYK fermentation broth (1 × 10^9^ CFU ml^-1^) was centrifuged for 30 min at 12,000 rpm. SnebYK filtrate (1 ml) was added to each well of a 12-well tissue culture plate containing 1 ml suspension of freshly hatched *H. glycines* J2 (35–50 juveniles ml^-1^, surface-sterilized). Filtrate of *B. megaterium* fermentation broth (1 × 10^9^ CFU ml^-1^), which showed high contact toxicity to *H. glycines* J2 in our previous work (Zhou et al., 2017), was used as a positive control; sterile water was used as a negative control. The plate was incubated in the dark at 25 ± 2°C. Immotile J2 were considered dead when they were needled without reaction (Cayrol et al., 1989). The number of dead J2 was counted under a microscope (SMZ800, Nikon, Japan) after 12 and 24 h of incubation. Each treatment included three replicates, and the experiment was repeated three times.

#### Potting and Field Experiments

Soybean seeds were surface-sterilized and coated with SnebYK as described above, and the control seeds were coated with sterilized water. When soybean seedlings grew to the two-euphylla stage, their roots were inoculated with 2,000 J2 of *H. glycines*. Thirty days after nematode inoculation, the soybean roots were harvested, and the adult female nematodes on the entire root surface were counted. The roots were then washed with tap water, stained with NaClO-acid fuchsin, and the number of *H. glycines* in each root was counted (Bybd et al., 1983). Adult females and nematodes in the soil were screened by sieving and counted under a stereomicroscope (Liu, 1995). To ascertain the effect of SnebYK on soybean seedlings growth under *H. glycines* stress, the shoot length, taproot length, and fresh weight of seedlings were measured. The root morphology was analyzed using the WinRHIZO image analysis system as previously described. In each case, there were seven replicates, and the entire experiment was repeated twice.

Field trials were carried out in a field infested with *H. glycines* at the experimental test site of the Modern Agricultural Industry Technology System of China in Kangping, located at 42°43′N, 123°24′E, during the growing seasons of 2016 and 2017. Soybean seeds were coated with SnebYK or sterile water in a 70:1 mass ratio. Seeds for each treatment were then sown in six rows of 70 seeds with 10 cm intervals between seeds, in a 3.5 m × 7 m plot. A randomized complete block design was adopted in this experiment, and each treatment included five plots. Thirty days after sowing, 12 seedlings were randomly selected from the inner four rows of each plot and nematode infections were evaluated. Adult females of *H. glycines* on the roots and in the rhizosphere soil (100 ml), and the nematodes in the roots, were counted as described above. The shoot length, taproot length, and fresh weight of the soybean seedlings were also measured. The field experiment was conducted in a fenced test field kept separate from animals and other crops.

### Split Root Experiment

**FIGURE 1:**
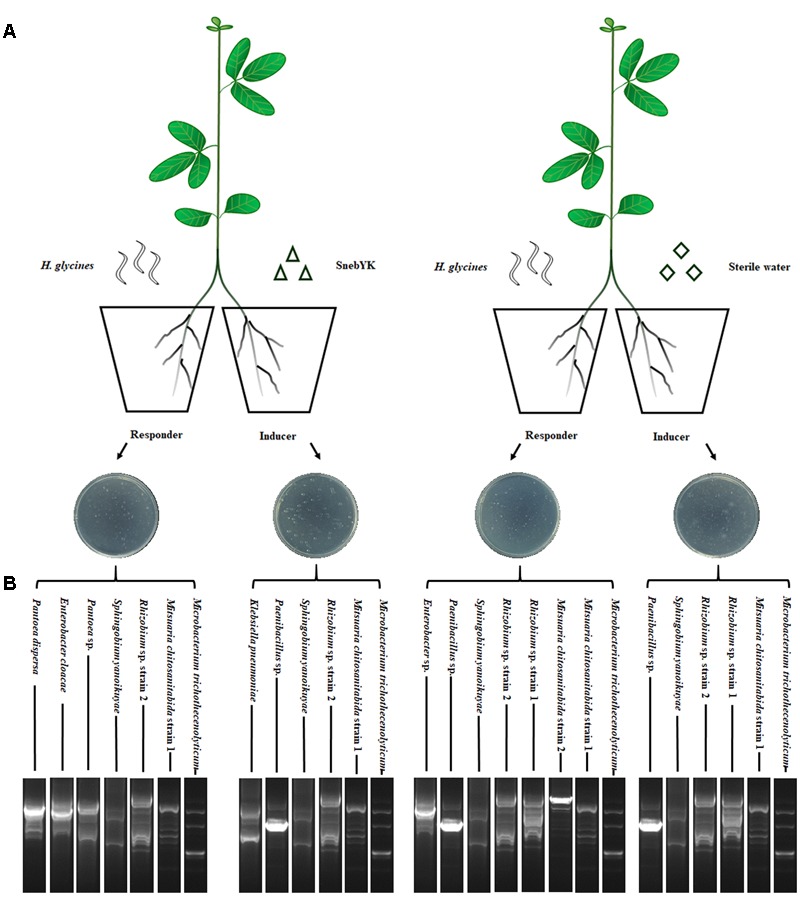
Split root system used to study induced systemic resistance against *Heterodera glycines* in soybean. **(A)** SnebYK and *H. glycines* were inoculated into opposite pots containing the inducer and responder root systems, respectively. Controls were inoculated with sterile water. **(B)** BOX-PCR fingerprints and 16S rRNA identification of rhizosphere/endophytic bacteria in the split root system.

Soybean seeds were surface-sterilized as described and sown in pots containing the sterilized soil mixture, with one seed per pot. When soybean seedlings had two euphylla, the roots were removed from the mixture and washed thoroughly with running tap water. Roots of approximately the same size were selected and divided into two equal parts with a scalpel: one part was designated the inducer root system, and the other part was designated the responder root system (Adam et al., 2014). The inducer and responder roots were transplanted into two plastic pots, with 200 ml of sterilized mixture in each. When the two parts of the root systems had been successfully fixed, the inducer root system was inoculated either with SnebYK fermentation broth (10 ml, 1 × 10^9^ CFU ml^-1^) or with sterile water as a control. After 5 days of treatment, all responder roots were inoculated with 2,000 J2 of *H. glycines* as shown in **Figure 1A**. Fifteen days after nematode inoculation, the responder roots were stained with NaClO-acid fuchsin (Bybd et al., 1983). The number of *H. glycines* in the roots was counted, and the developmental stages of *H. glycines* were recorded simultaneously. Each treatment had four repetitions, and the entire experiment was repeated twice.

To verify the validity of the split root system (SRS), BOX-PCR analysis and 16S rRNA gene sequencing were performed to test the colonization of the root system by SnebYK. Twenty days after the inducer roots were inoculated with SnebYK or sterile water, the responder and inducer root systems were removed. Immediately, 0.5 g of each root was collected and ground with a tissue grinder to release the rhizosphere and endophytic bacteria. A bacterial suspension was obtained after adding 1 ml of sterile water to the tissue grinder. After further dilution with sterile water (1,000×), 100 μl of the suspension was spread onto an ACCC55 agar plate and incubated at 28°C. Each treatment included three replications, and all steps were conducted under sterile conditions. After 72 h, morphologically distinct colonies of bacteria were selected and cultured separately. The genomic DNA of each strain was extracted with a bacterial genomic DNA extraction kit (Tiangen Biotech, China). The electrophoretograms of the BOX-PCR products were used to screen out different bacterial strains and could also be used to investigate the colonization of SnebYK by comparing them with the SnebYK electrophoretogram. The primer used for the BOX-PCR was BOXA1R (5′-CTACGGCAAGGCGACGCTGACG-3′) (Versalovic et al., 1994). The amplification was conducted following the method reported by Ateba and Mbewe (2013) in a 25 μl reaction mixture with the DNA samples obtained from the bacterial strains found in the SRS. Strains with different fingerprinting profiles were identified by 16S rRNA gene sequencing as described by Sarma and Saikia (2014).

### RNA Isolation and Real-Time PCR Analysis of Gene Expression

Soybeans were treated with SnebYK and grown as previously described for the pot experiment. Sterilized water was used as control. When soybean seedlings reached the two-euphylla stage, their roots were inoculated with 2,000 J2 of *H. glycines*. To measure the transcript levels of defense-related genes in real time, soybean roots were collected at 0, 24, and 48 h after nematode inoculation, froze immediately in liquid nitrogen and stored at -80°C until use. Total RNA was extracted from the soybean roots using the MiniBEST Universal RNA Extraction kit (TaKaRa, China). Total RNA from each sample (1 μg) was reverse-transcribed using the PrimeScript RT Reagent kit (TaKaRa, China). Quantitative real-time PCR (qRT-PCR) was performed in a CFX Connect Real-Time PCR Detection System (Bio-Rad, United States) using a SYBR green I quantitative PCR master mix (TaKaRa, China). The reaction conditions were 95°C for 30 s followed by 40 cycles at 95°C for 5 s, and 60°C for 30 s (fluorescence measurement step). After 40 cycles, a melting-curve analysis was performed (15 s at 95°C, 30 s at 60°C, and 15 s at 95°C, fluorescence measurement step). Each sample included three replicates. Expression of the soybean gene *Actin11* was used as an internal standard for normalization, and the data were quantified using the 2^-ΔΔCt^ method (Livak and Schmittgen, 2001).

### Statistical Analysis

All data were analyzed using SPSS Statistics 19.0 (**RRID**: *SCR_002865*) and expressed as the mean ± standard deviation. Significant differences among treatments in the *in vitro* juvenile mortality assay were determined according to Duncan’s multiple range test (*P* < 0.05). Significant differences between treatments in other experiments were evaluated using *t*-tests (*P* < 0.05).

## Results

### Plant Growth-Promoting Properties of SnebYK

**FIGURE 2:**
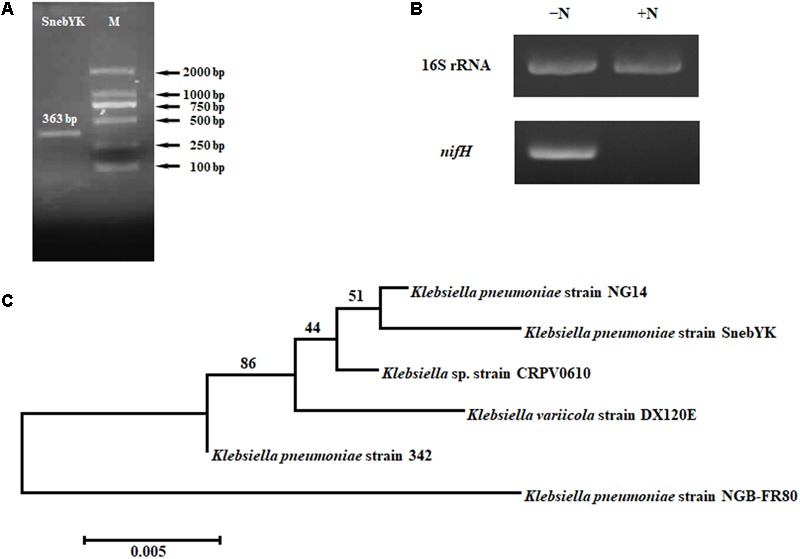
Amplification and analysis of the *nifH* gene of SnebYK. **(A)** Amplicon of SnebYK *nifH* (363 bp) in the left lane and DNA ladder in the right lane. **(B)** Transcriptional analysis of the SnebYK *nifH* gene in nitrogen-free medium determined with RT-PCR. The lane labeled “-N” shows the expression of the 16S rRNA and *nifH* gene of SnebYK grown in ACCC55 nitrogen-free medium; the lane labeled “+N” shows the expression of the 16S rRNA and *nifH* gene of SnebYK grown in nutrient agar medium. The transcript level of the 16S rRNA was used as a loading control; the transcript level of *nifH* of SnebYK grown in nutrient agar medium was used as a negative control. **(C)** Dendrogram based on the *nifH* sequences of SnebYK and other strains in the genus *Klebsiella* with similar *nifH* sequences. This analysis was performed using the neighbor-joining method in Mega 7.0.26 with a bootstrap value of *n* = 1000.

*Klebsiella pneumoniae* has been reported to possess many plant growth-promoting properties. Therefore, several such properties, including nitrogen fixation, ammonia production, phosphate solubilization, and siderophore production, were analyzed in SnebYK (**Table 1**). An orange halo zone was observed on the chrome azurol S agar plate, indicating that SnebYK produced siderophores. The ability of SnebYK to solubilize inorganic phosphate was confirmed by the clear zone around SnebYK on the National Botanical Research Institute’s phosphate agar plate. However, nothing was observed on the Mongina agar plate, which indicates that SnebYK could not solubilize organic phosphate. SnebYK grown on the nitrogen-free medium showed high nitrogenase activity. The yellow color observed following the addition of Nessler’s reagent to culture filtrate indicated that SnebYK could produce ammonia. A 363 bp amplicon specific to *nifH* (GenBank accession number: MF580385), a functioning gene encoding nitrogenase iron protein, was obtained from the genomic DNA of SnebYK (**Figure 2A**). The RT-PCR results showed that the *nifH* gene was expressed (1.24-fold the level of the internal control) when SnebYK was cultured under nitrogen-free conditions but was not expressed when SnebYK was cultured in a medium with sufficient nitrogen (**Figure 2B**). Phylogenetic analysis revealed that the *nifH* sequence of SnebYK was closely related to that of the *K. pneumoniae* strain NG14 and other strains in the genus *Klebsiella* (**Figure 2C**).

**Table 1:** Plant growth-promoting properties of SnebYK.

Properties	Activity^a^	Concentration^b^
Inorganic phosphate solubilization	+	0.14 ± 0.00
Organic phosphate solubilization	-	-
Ammonia production	+	258.39 ± 35.13
Siderophore production	+	49.83 ± 1.45
Nitrogen fixation	+	2.60 ± 0.13

*^*a*^Positive result (+); negative result (-). ^*b*^The values are means ± SD (*n* = 3); -, no data available due to a lack of activity; nitrogen fixation ability is expressed as μmol C_*2*_H_*4*_/h/10^**9**^ cells; other values are expressed as μg ml^-*1*^.*

### Effect of SnebYK on the Growth of Soybean Seedlings

**FIGURE 3:**
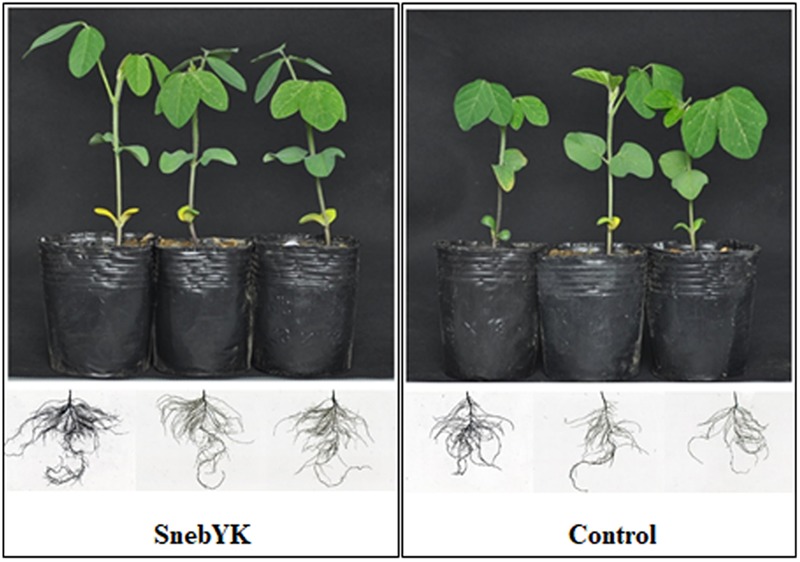
Effect of SnebYK on the aboveground and belowground growth of soybean.

Since SnebYK exhibits a variety of growth-promoting properties, the effect of SnebYK on soybean growth was evaluated. SnebYK-treated soybean plants had well-developed second compound leaves and roots (**Figure 3**). The second compound leaves of untreated soybean were not fully expanded, and their roots were sparse. In the presence of SnebYK, soybean seedlings exhibited increased biomass (**Table 2**). SnebYK caused significant increases in the shoot length and fresh weight, which were 9.18% and 39.51% higher, respectively, than those in the control. However, SnebYK did not affect taproot length or the root–shoot ratio. SnebYK also had a considerable impact on root growth. SnebYK increased the total root length, total root surface area, and total root volume by 52.10%, 47.73%, and 42.72%, respectively, compared to those of the untreated control.

**Table 2:** Effect of SnebYK on the growth of soybean seedlings.

Parameters	Treatments	
	SnebYK	Control
Shoot length (cm)	11.30 ± 0.31a	10.35 ± 0.27b
Taproot length (cm)	33.65 ± 2.69a	25.70 ± 3.25a
Total root length (cm)	1031.35 ± 43.52a	678.09 ± 53.43b
Total root surface area (cm^2^)	194.91 ± 7.50a	131.94 ± 9.25b
Total root volume (cm^3^)	2.94 ± 0.12a	2.06 ± 0.14b
Fresh shoot weight (g)	1.76 ± 0.07a	1.27 ± 0.05b
Fresh root weight (g)	3.36 ± 0.11a	2.40 ± 0.14b
Fresh seedling weight (g)	5.12 ± 0.15a	3.67 ± 0.17b
Root–shoot ratio	1.93 ± 0.08a	1.88 ± 0.08a

*The values are means ± SD (*n* = 10). Different letters (a, b) following the values indicate significant differences between the treatments as determined by a *t*-test (*P* < 0.05).*

### Effect of SnebYK on the Control of *H. glycines* in the Pot and the Field

**FIGURE 4:**
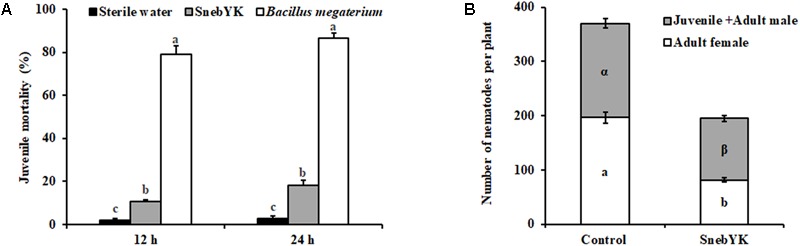
Effects of SnebYK on the control of *Heterodera glycines in vitro* and in the potting experiment. **(A)** *In vitro* juvenile mortality of *H. glycines* at 12 and 24 h after treatment with culture filtrates of SnebYK. Sterile water was used as a negative control; *Bacillus megaterium* was used as a positive control. The error bars represent standard deviations, and different letters (a, b) above the bars indicate significant differences at *P* < 0.05 according to Duncan’s multiple range test (*n* = 3). **(B)** Effect of SnebYK on the control of *H. glycines* in pots following seed coating. The numbers of *H. glycines* in the roots and soil were counted 30 days after inoculation. Different letters (a, b or α, β) indicate statistically significant differences (*t*-test with *P* < 0.05); a or b indicates a significant difference in the number of adult females, and α or β indicates a significant difference in the number of juveniles + adult males between treatments.

SnebYK had low contact toxicity to *H. glycines*, as the mortality rate after 24 h was only 18.23% (**Figure 4A**). To evaluate whether coating seeds with SnebYK provided protection against *H. glycines*, seeds were sown in pots after coating and then the soybeans were inoculated with *H. glycines*. The number of females significantly influences the nematode population. Thus, we evaluated the effect of SnebYK on the control of both the adult female and the total population of *H. glycines*. Thirty days after *H. glycines* inoculation, juveniles and adults, especially adult females, in the soil and the soybean roots were counted. The nematode population of SnebYK-treated soybean was significantly smaller than that of the control, with inhibition rates reaching 47.32% (**Figure 4B**). Analysis of the population revealed that SnebYK dramatically decreased the numbers of both juveniles and adult males, as well as adult females. Interestingly, the proportion of adult females in the *H. glycines* population was 40.88% (±4.77%) after treatment with SnebYK, while that in the control was 52.42% (±2.19%). This indicates that SnebYK significantly decreased the proportion of adult females, demonstrating that SnebYK effectively restrained the population of *H. glycines* and prevented their development into females. In the presence of *H. glycines*, SnebYK-treated soybean maintained a higher fresh root weight than the untreated control, concomitantly with a significantly increased root–shoot ratio (**Table 3**). After analysis of the root system, we found that the total root volume of SnebYK-treated soybean was significantly higher, by 28.74%, than that in the control (**Table 3**).

**Table 3:** Effects of SnebYK on the growth of soybean seedlings in the presence of *Heterodera glycines*.

Parameters	Treatments	
	SnebYK	Control
Shoot length (cm)	30.71 ± 1.16a	32.29 ± 1.83a
Taproot length (cm)	25.14 ± 1.74a	24.36 ± 2.29a
Total root length (cm)	581.31 ± 40.22a	480.42 ± 51.22a
Total root surface area (cm^2^)	90.46 ± 6.85a	72.09 ± 6.01a
Total root volume (cm^3^)	1.12 ± 0.10a	0.87 ± 0.05b
Fresh shoot weight (g)	2.10 ± 0.14a	2.21 ± 0.13a
Fresh root weight (g)	1.58 ± 0.12a	1.27 ± 0.06b
Fresh seedling weight (g)	3.68 ± 0.22a	3.48 ± 0.18a
Root–shoot ratio	0.76 ± 0.05a	0.58 ± 0.03b

*The values are means ± SD (*n* = 7). Different letters (a, b) following the values indicate significant differences between the treatments as determined by a *t*-test (*P* < 0.05).*

**FIGURE 5:**
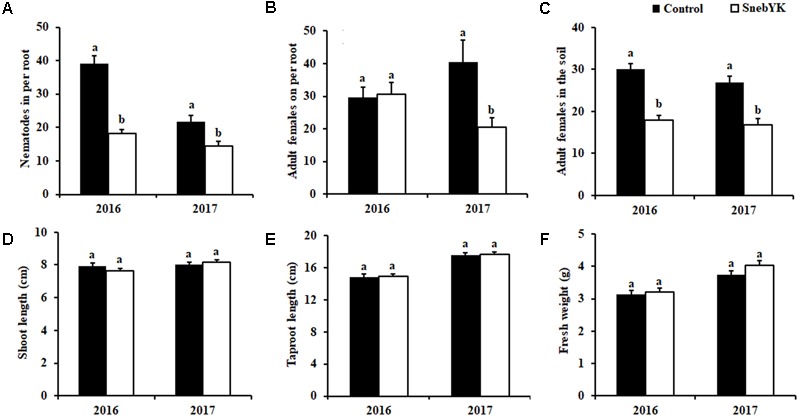
Effects of SnebYK on the control of *Heterodera glycines* and on soybean growth in the field following soybean seed coating. **(A)** Effect of SnebYK on the control of *H. glycines* in soybean roots. **(B)** Effect of SnebYK on the control of adult females of *H. glycines* on soybean roots. **(C)** Effect of SnebYK on the control of adult females of *H. glycines* in 100 ml rhizosphere soil. **(D)** Effect of SnebYK on the shoot length of soybean infested with *H. glycines* in the field. **(E)** Effect of SnebYK on the taproot length of soybean infested with *H. glycines* in the field. **(F)** Effect of SnebYK on the fresh weight of soybean infested with *H. glycines* in the field. Error bars represent standard deviations, and different letters (a, b) above the bars indicate significant differences at *P* < 0.05 according to a *t*-test (*n* = 60).

To determine the effect of SnebYK on the control of *H. glycines* in the field, experiments were conducted at the experimental test site in Kangping. We observed that SnebYK effectively reduced the number of *H. glycines* in per root by 53.23% and 33.75% in 2016 and 2017, respectively (**Figure 5A**). The 2016 data showed that SnebYK did not reduce the number of adult females on the soybean roots but did reduce their abundance in the soil by 40.11%. The 2017 data showed that SnebYK greatly reduced the number of adult females both on the roots and in the soil (**Figures 5B,C**). **Figures 5D–F** summarize the effect of SnebYK on soybean growth under *H. glycines* infection in the field trials. The results indicated that, although SnebYK-treated soybeans had a higher fresh weight than that of the untreated soybeans, the difference was not significant.

### Effect of SnebYK on Inducing Systemic Resistance to *H. glycines* in Soybean

**FIGURE 6:**
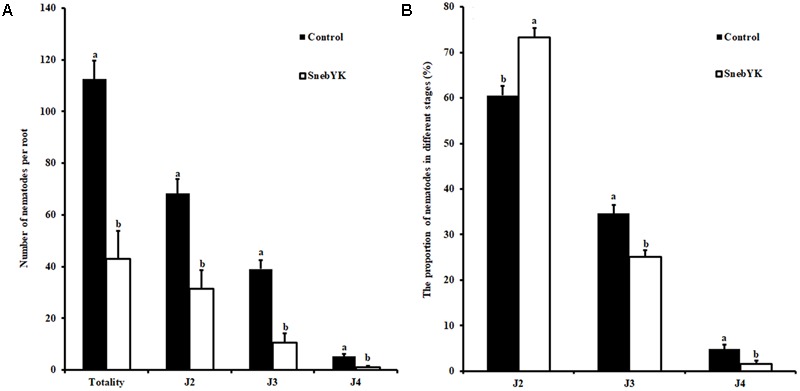
Effect of SnebYK on inducing systemic resistance to *Heterodera glycines* using a split root system in soybean. **(A)** Effect of SnebYK on the penetration and development of *H. glycines* in the responder root system. **(B)** Effect of SnebYK on the proportion of each developmental stage of *H. glycines* in the responder root system. Numbers of *H. glycines* in responder roots were counted 15 days after inoculation. J2, J3, and J4 indicate second-, third-, and fourth- stage juveniles of *H. glycines*, respectively. Error bars represent standard deviations, and different letters (a, b) above the bars indicate significant differences at *P* < 0.05 according to a *t*-test (*n* = 4).

While SnebYK resulted in low *H. glycines* mortality in the *in vitro* assay, it exhibited the capacity to control *H. glycines* in both the potting and field experiments when used to coat soybean seeds. Therefore, we conducted experiments to verify whether SnebYK could elicit systemic resistance in soybean against *H. glycines*. The SRS is considered an effective tool for demonstrating induced resistance, and in this study, we confirmed the validity of the SRS before using it. Rhizosphere and endophytic bacteria from the SRS were cultured on ACCC55 agar plates. After 1 day, several bacterial colonies (with morphologies similar to that of SnebYK) were observed on only the agar plate that was inoculated with the bacteria from the inducer root treated with SnebYK. More bacterial colonies appeared after 2 days. When comparing the BOX-PCR fingerprints and 16S rRNA identifications of rhizosphere and endophytic bacteria from the SRS, we found *K. pneumoniae* in only the inducer roots treated with SnebYK (**Figure 1B**). Since the responder roots were not colonized by SnebYK, the SRS used in this experiment was valid. Next, we conducted a split root experiment to determine whether SnebYK elicited ISR to *H. glycines* in soybean. Penetration of the responder roots of the SRS, assessed 15 days after *H. glycines* inoculation, was significantly reduced when the inducer roots of the soybean were treated with SnebYK. This treatment caused a 61.78% reduction in the total nematode penetration when compared with that in the untreated control. Specifically, the numbers of J2, third-stage juveniles (J3), and fourth-stage juveniles (J4) were significantly lower in the responder roots than in the control group (**Figure 6A**). The proportion of *H. glycines* juveniles of each stage in the responder roots is summarized in **Figure 6B**. The proportions of J2, J3, and J4 in the control group were 60.59%, 34.62%, and 4.8%, respectively. Meanwhile, the proportions in the SnebYK-treated group were 73.27%, 25.1%, and 1.63%, respectively. Therefore, inoculation with SnebYK significantly increased the proportion of J2 by 12.68% and decreased the proportions of J3 and J4 by 9.52% and 3.17%, respectively.

**FIGURE 7:**
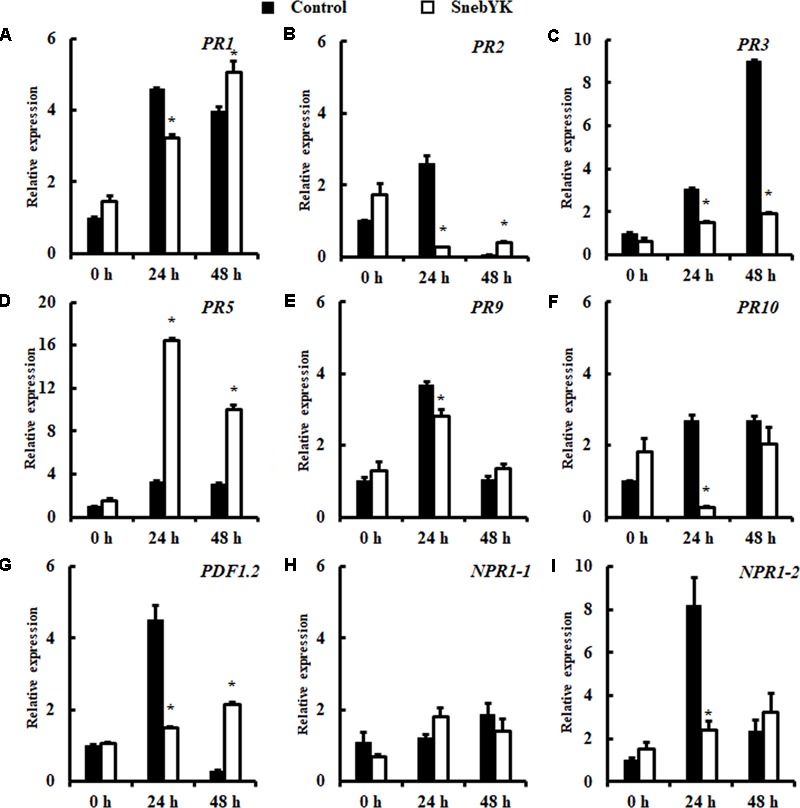
Induction of defense genes in soybean roots after *Heterodera glycines* inoculation. Soybean root samples were taken at 0, 24, and 48 h after *H. glycines* inoculation, and the expression levels of nine defense genes were analyzed by qRT-PCR: **(A)** *PR1*, **(B)** *PR2*, **(C)** *PR3*, **(D)** *PR5*, **(E)** *PR9*, **(F)** *PR10*, **(G)** *PDF1.2*, **(H)** *NPR1-1*, and **(I)** *NPR1-2*. Error bars represent standard deviations; a *t*-test was used to determine significant differences between the SnebYK-treated sample and the control (^∗^*P* < 0.05).

To investigate whether SnebYK-mediated ISR was accompanied by primed defense responses to *H. glycines* in the roots, the expression levels of *PR1, PR2, PR3, PR5, PR9, PR10, PDF1.2, NPR1-1*, and *NPR1-2* in soybeans treated or not treated with SnebYK were assessed using qRT-PCR. Transcript levels of the defense genes analyzed did not differ in the non-challenged soybeans regardless of the presence of SnebYK. After *H. glycines* inoculation, elevated *PR5* expression was detected in control roots, indicating that soybean responds rapidly to *H. glycines* infection. Moreover, transcription of *PR5* in soybeans pretreated with SnebYK was 5.04-fold and 3.23-fold higher at 24 and 48 h after inoculation, respectively, than those in control soybeans. Differences were also found in the expression levels of *PR1, PR2, PR3, PR9, PR10, PDF1.2*, and *NPR1-2* between untreated soybeans and soybeans pretreated with SnebYK upon challenge with *H. glycines*. Twenty-four hours after *H. glycines* inoculation, these genes were induced in control soybeans, whereas the increase upon *H. glycines* challenge was lower in soybeans previously treated with SnebYK. However, 48 h after inoculation of *H. glycines*, the expression levels of *PR1, PR2* and *PDF1.2* were significantly higher in soybeans pretreated with SnebYK than in control soybeans; the transcript levels of *PR9, PR10* and *NPR1-2* did not differ significantly between the treatments; and the expression of *PR3* was much lower in SnebYK-treated soybean roots than in untreated soybean roots (**Figure 7**). The transcript levels of *NPR1-1* did not differ between soybeans pretreated with SnebYK and soybeans not treated with SnebYK following the inoculation of *H. glycines*.

## Discussion

Soybean is an important legume, and its production is greatly threatened by *H. glycines*. PGPB provide one of the most efficient control strategies in modern agriculture. In this study, the effect of SnebYK on the control of nematodes was evaluated. Data collected in potting experiment demonstrated that the population of *H. glycines* in SnebYK-treated soybean roots decreased by 47.32%; concomitantly, the number of adult females was reduced by 58.56% compared to that in the control. The number of female nematodes has an important effect on population size (Triantaphyllou, 1973) and was therefore a focus of this study. The results of the field trials also confirmed the ability of SnebYK to control *H. glycines*. By coating soybean seeds, SnebYK not only reduced the number of *H. glycines* in the soybean roots but also suppressed the number of adult females. Our data showed that SnebYK could effectively delay the development of juvenile nematodes into adult females, hindering mating and reproduction and thereby decreasing the abundance of *H. glycines*. These results are consistent with previous reports of PGPB and other beneficial microbes (Martinuz et al., 2013; Séry et al., 2016; De Medeiros et al., 2017; Kumari, 2017; Zhou et al., 2017). However, we found that SnebYK exhibited only a weak ability to kill *H. glycines in vitro*. Therefore, we speculated that SnebYK prevented *H. glycines* by inducing resistance.

Although some studies have demonstrated that *K*. *pneumoniae* can elicit ISR to pathogenic fungi and phytotoxicity in plants (Luo, 2010; Ji et al., 2014), these studies employed seed treatment. By establishing a split root experiment, we gained better insight into *K*. *pneumoniae*-elicited induced resistance. In the present study, we designed experiments to confirm that no colonization of SnebYK was present in the responder root system, to guarantee that bioprotection occurred when SnebYK and *H. glycines* were spatially separated. Indeed, SnebYK in the inducer roots reduced the *H. glycines* population in the responder roots, which clearly demonstrates that SnebYK-mediated bioprotection was at least partially systemically induced. The results of the split root experiment showed that SnebYK effectively triggered ISR in soybean against *H. glycines* and resulted in significantly reduced penetration. Similar results have been observed in *R. etli* (Martinuz et al., 2012), *B. subtilis* (Adam et al., 2014), and *S. fredii* (Tian et al., 2014) in defense against nematodes. In the split root experiment, the proportion of J2 *H. glycines* in SnebYK-treated soybean was significantly higher than that in the untreated control; correspondingly, the proportions of J3 and J4 decreased substantially. This result suggests that SnebYK plays a prominent role in delaying the development of *H. glycines* via ISR. Martinuz et al. (2013) reported similar results following *R. etli* G12 treatment, which reduced *M. incognita* penetration, delayed J2 development to J3, and reduced the numbers of females and eggs. Plants accumulate pathogenesis-related (PR) proteins in response to various pathogens, such as fungi, bacteria, viruses, insects, and nematodes (Kitajima and Sato, 1999; Van Loon et al., 2006). During *H. schachtii* infection of *Arabidopsis*, the expression of *PR1, PR2*, and *PR5* was induced, and that of *PR3* and *PR4* was not altered in roots (Hamamouch et al., 2011). Non-expressor of pathogenesis related genes-1 (NPR1) is a transcriptional co-activator of *PR* gene expression (Dong, 2004), and transgenic tobacco plants constitutively expressing *AtNPR1* exhibited resistance to *M. incognita* (Priya et al., 2011). In our study, SnebYK-mediated resistance to *H. glycines* was also accompanied by boosts in the expression of defense genes. Forty-eight hours after inoculation with *H. glycines*, the transcript levels of *PR1, PR2, PR5*, and *PDF1.2* increased in SnebYK-pretreated soybeans compared with those in non-pretreated soybeans; however, the expression of *PR3* decreased. Plant resistance to pathogens involves the plant hormones SA, JA, and ET. *PR1, PR2*, and *PR5* are considered to be markers of SA-dependent signaling pathways (Delaney et al., 1994; Penninckx et al., 1996; Thomma et al., 1998), while *PR3, PR4*, and *PDF1.2* are marker genes for JA/ET-dependent signaling pathways (Penninckx et al., 1998; Thomma et al., 1998). The *PR* genes with significant expression differences in the present study are all marker genes in the SA or JA/ET signaling pathways, including *PR1, PR2, PR3, PR5*, and *PDF1.2*. ISR often relies on pathways regulated by JA/ET (Van Oosten et al., 2008; Pieterse et al., 2009). However, dependence on both SA and JA/ET signaling pathways has also been reported (Niu et al., 2011; Salas-Marina et al., 2011). Our results implied that SnebYK might also activate SA and JA/ET signaling pathways to induce plant defense against *H. glycines*.

Treatment of soybean seeds with SnebYK significantly improved soybean seedling growth, especially that of the root. Several mechanisms may contribute to the increase in biomass. SnebYK carries the nitrogen-fixing gene *nifH* and expresses it under nitrogen-deficient conditions. SnebYK fixes atmospheric nitrogen, as shown by its ability to reduce acetylene. Non-symbiotic N_2_-fixing bacteria interact with plants to provide available nitrogen, increasing their vegetative growth and yield (Elkoca et al., 2010; Hayat et al., 2010). *Klebsiella pneumoniae* 342 is well known for its nitrogen-fixing capabilities. When *K. pneumoniae* 342 was applied to wheat, the dry weight of the plant increased; when the *nifH* gene of this strain was knocked out, the growth-promoting effect disappeared (Iniguez et al., 2004). Several experiments have examined the effects of *K. pneumoniae*, as a non-symbiotic N_2_-fixing bacterium, on the growth of monocotyledonous plants, such as rice (Ji et al., 2014; Banik et al., 2016), maize (Kuan et al., 2016), and wheat (Remus et al., 2000). However, few data are available on the effects of *K. pneumoniae* on the dicotyledonous plants growth. Our results demonstrated that SnebYK exhibited growth-promoting effects on the dicotyledonous soybean. Soybean roots treated with SnebYK were larger than those of the controls. PGPB affect root morphology, particularly by increasing root surface area (Larraburu et al., 2007; Wang et al., 2015) and weight (Islam et al., 2016; Singh et al., 2017), thereby improving nutrient uptake. This mechanism is even more important than nitrogen fixation in some PGPB (Vessey, 2003). SnebYK produces large amounts of ammonia, which may play an important role in the suppression of nematode populations (Rodriguez-Kabana, 1986; Khan et al., 2012). Phosphorus is also a key nutrient element for plants. SnebYK can convert tricalcium phosphate to a form that is accessible to the host. SnebYK also produces siderophores, which block the proliferation of pathogenic microorganisms by chelating Fe^3+^ and act as one of the major determinants of ISR (Hiifte et al., 1994; Ramamoorthy et al., 2001). The main signaling steps during the development of ISR require gene expression and consume resources (Buell, 1999; Heil, 2001), and SnebYK treatment activated the expression of *PR* genes under *H. glycines* infection. Allocation of limited plant resources to defense, means they cannot be used for plant growth (Herms and Mattson, 1992; Heil, 2001). Our data indicated that SnebYK fixes nitrogen and solubilizes inorganic phosphate, implying that SnebYK might provide nutrition to plants. This additional nutrition might be a reason that SnebYK-treated soybean exhibited a higher biomass and resistance to *H. glycines* at the same time. Further experiments are required to explore the regulatory mechanism in detail.

## Conclusion

Our results support a role for the PGPB strain *K. pneumoniae* SnebYK in the induction of ISR against *H. glycines* in soybean. This study is the first report of *K. pneumoniae* eliciting ISR against *H. glycines*. SnebYK showed an ability to inhibit both the invasion and the development of nematodes. It induced the expression of defense genes in soybean following *H. glycines* infection and suppressed *H. glycines* in the field after coating soybean seeds, suggesting this may be an efficient and economical method to control *H. glycines*. SnebYK also presented remarkable plant growth-promoting properties, which promoted the growth of soybean. The overall results of the present study support *K. pneumoniae* SnebYK as a potential biocontrol agent for *H. glycines*.

## Author Contributions

DL, LC, and YD conceived and designed the research. DL, LC, XZ, YW, and YX conducted all experiments and data analyses. The manuscript was written by DL and YD. XL and LJC provided the instruments and critically revised the manuscript. YD provided the final approval of the version to be published. All authors read and approved the final manuscript.

## Conflict of Interest Statement

The authors declare that the research was conducted in the absence of any commercial or financial relationships that could be construed as a potential conflict of interest.
